# Efficacy of a commercial herbal formula in chicken experimental coccidiosis

**DOI:** 10.1186/s13071-019-3595-4

**Published:** 2019-07-12

**Authors:** Loredana Maria Pop, Erzsébet Varga, Mircea Coroian, Maria E. Nedișan, Viorica Mircean, Mirabela Oana Dumitrache, Lénárd Farczádi, Ibolya Fülöp, Mircea Dumitru Croitoru, Mihaly Fazakas, Adriana Gyӧrke

**Affiliations:** 1 Department of Parasitology and Parasitic Diseases, Faculty of Veterinary Medicine, University of Agricultural Sciences and Veterinary Medicine Cluj-Napoca, Calea Mănăştur 3–5, 400372 Cluj-Napoca, Romania; 2 Department of Pharmacognosy and Phytotherapy, Faculty of Pharmacy, University of Medicine, Pharmacy, Sciences and Technology of Târgu Mureș, 38 Gheorghe Marinescu, 540139 Târgu Mureș, Romania; 3 Center for Advanced Medical and Pharmaceutical Research, Laboratory of Chromatography LC/MS, Faculty of Pharmacy, University of Medicine, Pharmacy, Sciences and Technology of Târgu Mureș, 38 Gheorghe Marinescu, 540139 Târgu Mureș, Romania; 4 Department of Toxicology and Biopharmacy, Faculty of Pharmacy, University of Medicine, Pharmacy, Sciences and Technology of Târgu Mureș, 38 Gheorghe Marinescu, 540139 Târgu Mureș, Romania

**Keywords:** *Eimeria*, Herbal extract, Broiler chickens, Anticoccidial effect, Polyphenols, LC-MS/MS

## Abstract

**Background:**

Coccidiosis represents a serious threat to the poultry industry, affecting production and causing high morbidity, mortality and significant costs resulting from treatment and prophylaxis. In-feed anticoccidials have been used for decades for managing avian coccidiosis and were very effective until drug resistance emerged. The use of natural remedies has become a promising alternative in combating coccidiosis in chickens. Therefore, the purpose of the present study was to assess the efficiency of a commercial herbal formula (H), as oral liquid preparations, in experimental chicken coccidiosis.

**Methods:**

Two independent controlled battery experiments (BE1 and BE2) were designed and the product was tested in 3 different formulas (H1, H2 and H3): H1 contained a propylene glycol extract of *Allium sativum* and *Thymus serpyllum*; H2 contained *Origanum vulgare*, *Satureja hortensis* and *Chelidonium majus*; and H3 contained *Allium sativum*, *Urtica dioica*, *Inula helenium*, *Glycyrrhiza glabra*, *Rosmarinus officinalis*, *Chelidonium majus*, *Thymus serpyllum*, *Tanacetum vulgare* and *Coriandrum sativum*. Chickens were divided into five groups for each BE as follows: (i) uninfected untreated control (UU1, UU2); (ii) infected untreated control (IU1, IU2); (iii) infected treated with amprolium (ITA1, ITA2); and (iv, v) two experimental groups infected treated with H1 (ITH1) and H2 (ITH2) formulas in the BE1 and with H3 (ITH3-5 and ITH3-10) formula in the BE2. The chickens from infected groups were challenged with 5000 (BE1) and 50,000 (BE2) sporulated oocysts of *Eimeria* spp. (*E. acervulina*, *E. tenella* and *E. maxima*), respectively. The anticoccidial efficacy was assessed by recording the following: oocysts output (OPG), lesion score (LS), weight gain (WG), feed conversion ratio (FCR) and anticoccidial index (ACI). Additionally, polyphenolics and flavonoids (caffeic-chlorogenic acid, apigenin, kaempferol, luteolin, quercitin, quercitrin) from herb extracts found in H3 formula were determined by the liquid chromatography-tandem mass spectrometry (LC-MS/MS) method.

**Results:**

H1 and H2 reduced the WG, and increased the FCR and OPG compared with controls. H1 reduced the duodenal lesions, whilst H2 reduced the caecal lesions, compared with control. H3 decreased the OPG of *Eimeria* spp., reduced the total lesion score and improved the zootechnical performance (weight gain and feed conversion ratio). According to ACI value, H1 and H2 had no efficacy on *Eimeria* spp. infection, but H3 had good to marked anticoccidial effect, the ACI being slightly greater in the group ITH3-5. According to the results of LC-MS/MS, the concentration of polyphenols in H3 formula was the highest, the sum of chlorogenic acid and caffeic acid being 914.9 µg/ml.

**Conclusions:**

H3 formula is a promising natural anticoccidial and field trials are recommended in order to validate the obtained data.

**Electronic supplementary material:**

The online version of this article (10.1186/s13071-019-3595-4) contains supplementary material, which is available to authorized users.

## Background

Coccidiosis is a parasitic disease caused by seven species of the genus *Eimeria* with different localizations within the intestinal tract of chickens. *Eimeria acervulina*, *E. maxima* and *E. tenella* are the most prevalent species in broilers in the intensive poultry management system [1, 2]. The disease represents a serious threat for the poultry industry, affecting the production, and causing high morbidity, mortality and significant economic loss due to the associated costs of treatment and prophylaxis. Global financial losses due to coccidiosis have been estimated at three billion USD per annum [3]. In-feed anticoccidials have been used for decades for managing avian coccidiosis and they were very effective until drug resistance emerged. To date, *Eimeria* strains have gained resistance to all known coccidiostats, and new anticoccidials are unlikely to be developed, mainly because of strict legislative regulations on the use of in-feed drugs and growing concerns in the general population about the chemical residues in poultry products [4–6]. Over the past years, the consumption of poultry meat has grown consistently, especially because it represents a fairly cheap source of food with lower production costs and accepted by all religions [7]. There is also a higher interest from the consumer in organic poultry production and a great demand for natural and healthier products [8]. In this context, the use of natural remedies has become a promising alternative to anticoccidial drugs [9]. Numerous plant-based products have been found to be effective at treating chicken coccidiosis: *Artemisia annua* and artemisinin [10, 11], oregano [12], garlic [13], neem [14], different species of *Aloe* [15], green tea [16], sugar cane [17], turmeric [18] and many others [9, 19–21]. Additionally, commercially available herbal combinations are already used in some countries for coccidiosis control [19]. Most of these natural compounds do not always aim directly at the parasites but have immunomodulatory effects, antioxidative or anti-inflammatory properties and act on the intestinal tract, thus helping the host organism to fight against the coccidial infection [9, 19]. Moreover, the plant extracts can have a direct effect on the parasites, by altering the process of oocyst wall formation and inhibiting sporulation [22, 23], or by destroying the sporozoites [24]. Furthermore, there is a lower risk of developing resistance to these natural substances compared to anticoccidial drugs [21]. Furthermore, herbal extracts could improve recovery after coccidiosis [25, 26]. Flavonoids and other polyphenols have been reported to be responsible for most of the biological properties of the herbs, including the anticoccidial potential [27].

Therefore, the purpose of the present study was to assess the effect of a commercial multi-plant extract compound, in experimental coccidiosis in broiler chickens. The composition of the herbal extract was designed on the basis of a literature search for effective anticoccidial natural compounds.

## Methods

### Animals and experimental design

Two independent controlled battery experiments (BE) were designed in order to assess the efficacy of a commercial herbal product (H), in three different formulas of propylene glycol/alcoholic herbal extracts. In the first BE (BE1), the anticoccidial effect of the first (H1) and second (H2) formulas was evaluated. The third formula (H3) was evaluated during the second BE (BE2).

One hundred one-day-old ROSS 308 hybrid broiler chickens were purchased from S.C. VIS AVIS S.A. (Vadu Crişului, Bihor, Romania) for each of the two BE. They were housed in batteries in dedicated facilities at the University of Agricultural Sciences and Veterinary Medicine Cluj-Napoca. At 14-days-old, broiler chickens were randomly divided in five groups, each with three replicates of five chickens/cage (*n *= 15). In both BE the experimental groups were represented by: (i) negative control, uninfected and untreated (UU1, UU2); (ii) positive control, infected and untreated (IU1, IU2); (iii) treatment control, infected and treated with amprolium (ITA1, ITA2); and (iv, v) two experimental groups, infected and treated with H1 (ITH1) and H2 (ITH2) formulas in the BE1and with H3 (ITH3-5 and ITH3-10) formula in the BE2.

On the same day, the broiler chickens were experimentally infected by the crop-route using insulin syringe, with 1 ml of a mixed suspension of fresh sporulated oocysts (BE1 5 × 10^3^ oocysts/chicken; BE2 5 × 10^4^ oocysts/chicken) containing *E. acervulina*, *E. tenella* and *E. maxima.* The strains were isolated in 2012 from a broiler farm and the species were identified by PCR [28]. The number of oocysts per milliliter was determined using a Fuchs-Rosenthal chamber and adjusted according to sporulation rate.

The control and experimental treatments were given *via* drinking water from 14 until 24 days of age, *ad libitum*. Chickens from ITA1 and ITA2 groups were treated with amprolium (Amprolium 20%^®^ water-soluble powder; Romvac Company S.A., Voluntari, Ilfov, Romania) as doses of 2.5 (BE1) and 5 g/l water (BE2), respectively. H1 and H2 herbal formulas were given as doses of 10 ml/l drinking water (BE1) and H3 herbal formula as doses of 5 (ITH3-5) and 10 (ITH3-10) ml/l drinking water (BE2).

The chickens were fed with standard starter (1–13 days-old) and grower (14–24 days-old) feed free of anticoccidials.

### Herbal formulas

The commercial herbal formula was supplied by S.C. PROMEDIVET S.R.L (Sovata, Mureș, Romania) as oral liquid preparations. The anticoccidial efficacy in chicken coccidiosis was evaluated for three different extracts named H1, H2 and H3. The extracts were obtained from grounded dried plants after maceration in propylene glycol (20 kg of dried plants in 120 l of propylene glycol) for 14 days and cold pressing.

H1 contained extracts from bulbs of *Allium sativum* (garlic) and leaves of *Thymus serpyllum* (wild thyme) in equal proportions. H2 was a mixture of extracts from leaves of *Origanum vulgare* (oregano) 40%, *Satureja hortensis* (summer savory) 30% and *Chelidonium majus* (greater celandine) 30%. H3 contained extracts from nine herbs: roots of *Urtica dioica* (nettle) 10%, *Inula helenium* (elecampane) 15%, *Glycyrrhiza glabra* (licorice) 10%, bulbs of *Allium sativum* 10%, leaves of *Rosmarinus officinalis* (rosemary) 10%, *Chelidonium majus* 10%, *Thymus serpyllum* 15%, flowers of *Tanacetum vulgare* (tansy) 10% and seeds of *Coriandrum sativum* (coriander) 10%.

### Liquid chromatography tandem mass spectrometry

The H3 formula was analyzed by liquid chromatography tandem mass spectrometry (LC-MS). One hundred microliters of H3 formula was mixed with 900 µl of purified water, then centrifuged at 10,000× *rpm* for 10 min and filtered using nylon micro pore 0.45 µm filters. Solutions were transferred to HPLC vials and 5 μl of the obtained samples were injected into the LC-MS system.

Chromatographic separation of analytes was performed using a NUCLEODUR C18 Gravity, 3 µm, 150 × 3 mm (Macherey-Nagel, Düren, Germany) with a mobile phase consisting of 0.2% formic acid in water and methanol in gradient elution, with a flow rate of 0.6 ml/min. Detection was carried out in multiple reactions monitoring mode (SRM). Ionization of analytes was performed using negative electrospray ionization mode. Ionization parameters used for the ionization source were as follows: spray voltage, 2500 V; vaporizer temperature, 350 °C; ion gas source 1, 25; ion gas source 2, 25; curtain gas, 10; declustering potential, 100; ion release delay, 30; ion release width, 15. Sample run-time was 30 min per sample.

Standard solutions of each analyte (chlorogenic acid, caffeic acid, quercitrin, luteolin, quercetin, apigenin and kaempferol) (100 µg/ml) were prepared in methanol. For analysis, standard solutions were mixed in methanol to a final concentration of 10 µg/ml for each analyte. Furthermore, the mixture was diluted to the following concentrations: 0.1 (lower limit of quantification, LLOQ), 0.25, 0.5, 1 and 10 µg/ml. The calibration of standard curve solutions was prepared by diluting 100 µl of each standard solution with 900 µl of purified water.

### Anticoccidial efficacy evaluation

The efficacy of the herbal formulas in experimental coccidiosis in broiler chickens was assessed by recording and calculating the number of oocysts shedded per gram of feces (OPG), lesion score (LS), mortality rate (MR), body weight gain (BWG), feed conversion ratio (FCR) and anticoccidial index (ACI).

The feces were collected on days 5, 7 and 10, from all 3 cages/group individually and the OPG was determined by duplicate counts of duplicate fecal slurries from each cage by using the McMaster method [29]. The detection limit was 12 oocysts/g feces. The LS was assessed on day 7 post-infection (pi) for eight chickens per group by using the scoring system of Johnson & Reid [30], according to the severity of lesions in duodenum, jejunum plus ileum and caeca. Chickens were weighed individually at the beginning of the experiments and at 7 days post-infection in order to calculate the body weight gain. The amount of feed given to the chickens was weighed daily for each cage, in order to calculate FCR, as the ratio between the amount of feed consumed per body weight gain of the chickens.

The ACI was calculated after the formula: ACI = (%S + %RGW) − (LI + OI) [31], where %S is the percentage of survival, %RGW is the percentage of relative weight gain (RWG = BWG × 100/untreated group BWG), LI is the lesion index as the lesion score multiplied by 10 and OI is the oocyst index as (OPG output of each experimental group/OPG output of the infected-unmedicated control) × 100. The interpretation of the results was made as follows: “lack of anticoccidian activity” when the value was lower than 120, “partially effective” at values of 120–160 and “very effective” at values higher than 160 [31].

### Statistical analysis

The data were processed with MedCalc Software v.18 (MedCalc Software bvba, Ostend, Belgium; https://www.medcalc.org). The arithmetic mean and standard error were calculated for each assessed parameter and for each group. ANOVA (repeated measures analysis of variance) was used for OPG and body weight gain, and the Kruskal-Wallis test was used for lesion score. Differences were considered statistically significant if the *P* ≤ 0.05. The experimental groups were compared with both positive and negative control groups and also with group A.

## Results

### First battery experiment

**Fig. 1:**
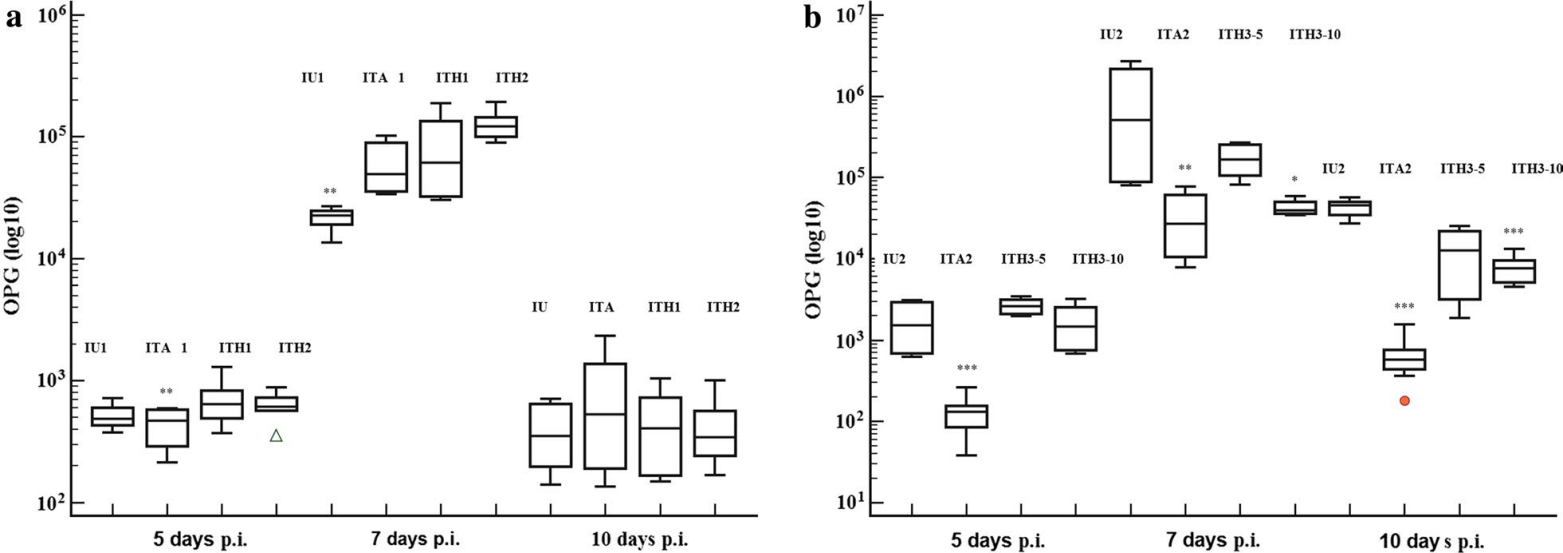
Dynamics of mean oocysts number/g of feces in experimental groups of chickens infected with *Eimeria* spp. (*E. acervulina*, *E. maxima* and *E. tenella*) and treated with the herbal product in different compositions compared with positive and amprolium control groups. **a** First battery experiment when chickens were infected with 5 × 10^3^ sporulated oocysts of *Eimeria* spp. and treated with H1 and H2 formulas, 10 ml/l water. **b** Second battery experiment when chickens were infected with 5 × 10^4^ sporulated oocysts of *Eimeria* spp. and treated with H3 formula, 5 (ITH3-5) and 10 ml/l water (ITH3-10)

The OPG of groups treated with H1 and H2 formulas was significantly higher compared with the positive control (IU1) or ITA1 groups on day 7 post-infection (p.i.) (*F*_(3,11)_ = 28.13, *P* < 0.001) (Fig. 1). The mortality rate was 0 for all experimental groups. During the necropsy, lesions due to *Eimeria* spp. infection were identified in duodenum and caecum in all infected groups. In all experimental infected groups, the total lesion score recorded values below 1 and with no significant differences (*χ*^2^ = 2.556, *df*  = 3, *P* = 0.465) between groups (Table 1). Nevertheless, the total lesion score in ITH1 and ITH2 groups was lower than the positive control group and higher than in group ITA1.

**Table 1:** The effect of the herbal product H on lesion score and performance parameters in experimental groups of chickens challenged with *Eimeria* spp. (*E. acervulina*, *E. maxima* and *E. tenella*) compared with control groups

	Lesion score			BWG	FCR
	Duodenum	Caecum	Total		
BE1					
UU1	0	0	0	55.81 ± 2.54	1.97 ± 0.11
IU1	0.4 ± 0.2	0.5 ± 0.2	0.9 ± 0.3	54.51 ± 1.55	1.77 ± 0.19
ITA1	0.1 ± 0.1	0.1 ± 0.1	0.3 ± 0.2	53.57 ± 2.52	1.64 ± 0.31
ITH1	0	0.5 ± 0.2	0.5 ± 0.2	53.30 ± 0.83	1.99 ± 0.30
ITH2	0.4 ± 0.2	0.1 ± 0.1	0.5 ± 0.2	47.07 ± 1.74*	2.24 ± 0.08
BE2					
UU2	0	0	0	46.89 ± 2.68	1.58 ± 0.17
IU2	1.33 ± 0.56	1.0 ± 0.00	2.33 ± 0.56	34.91 ± 4.39	2.04 ± 0.13
ITA2	0.00 ± 0.00*	0.8 ± 0.20	0.8 ± 0.20*	42.36 ± 2.08	1.61 ± 0.02
ITH3-5	0.83 ± 0.54	0.33 ± 0.21*	1.17 ± 0.65	40.57 ± 1.92	1.78 ± 0.03
ITH3-10	0.83 ± 0.48	0.5 ± 0.22	1.33 ± 0.42	39.80 ± 2.83	1.78 ± 0.02

*P < 0.05, Mann-Whitney test (independent samples) (MedCalc)

*Abbreviations*: BE, battery experiment (5 × 10^3^ oocysts/chicken in BE1 and 5 × 10^4^ oocysts/chicken in BE2); UU1, 2, negative control group; IU1, 2, positive control group; ITA1,2, Amprolium® 20% (Romvac Company SA, Voluntari, Ilfov, Romania), soluble powder treated group; ITH1, ITH2, ITH3-5 and ITH3-10, experimental groups treated with the herbal product in different compositions, 10 ml/l water (ITH1, ITH2 and ITH3-10) and 5 ml/l water (ITH3-5); BWG, body weight gain; FCR: feed conversion ratio

H1 formula prevented the emergence of *E. acervulina* lesions in the duodenum, but had no therapeutic/prophylactic effect on *E. tenella*; the lesion score in the caecum was similar with the positive control group. H2 formula did not prevent the occurrence of duodenal lesions, but the lesion score in the caecum was lower than in the positive control group (*χ*^2^ = 1.588, *df* = 1, *P* = 0.208) (Table 1).

All experimental groups presented lower weight gains compared with the negative control group, with statistical significance in the case of group ITH2 (*F*_(4,9)_ = 7.8, *P* = 0.007) (Table 1). The lowest weight gain was recorded in the group treated with H2 formula (Table 1). The FCR of negative and experimental groups was higher comparing with positive control group (Table 1).

According to the anticoccidial index, the H1 and H2 formulas had no efficacy on *Eimeria* spp. infection.

### Second battery experiment

The OPG value was significantly higher in groups treated with H3 formula than in the group treated with amprolium but not compared with the positive control group (*F*_(3,7)_ = 19.7, *P* < 0.001) at 5 days p.i. On days 7 and 10 p.i., the OPG of chickens treated with H3 decreased under the values of the positive group, but significantly only on day 10 p.i. (*F*_(3,7)_ = 43.44, *P* < 0.001) (Fig. 1). The group ITA2 recorded the lowest value of OPG compared with positive control and H3 treated groups during the entire recording period (*F*_(3,23)_ = 6.63, *P* = 0.003).

No mortality was registered in any experimental group. The chickens from all groups, with the exception of the ITA2 group, presented lesions in the duodenum, but all chickens had lesions in the caecum. Neither 5 nor 10 ml/l water of H3 formula significantly reduced the duodenal lesions compared with the positive control group. H3 formula at a dose of 10 ml/l water significantly reduced the caecal lesions (*Z* = 2.162, *P* = 0.031). Additionally, the chickens treated with 5 ml H3/l drinking water had fewer lesions than the positive control group, but with no statistical significance (Table 1). The total lesion score was reduced significantly compared with the positive control group only in the case of the ITA2 group (*Z* = 2.106, *P* = 0.0352). However, the H3 formula at a dose of 10 ml/l water reduced the total lesion score by 50%.

All the experimental groups presented lower weight gains than the negative control group (*F*_(4,11)_ = 1.78, *P* = 0.182). However, compared with the positive control group, the chickens treated with H3 formula had higher weight gains and only slightly lower than the amprolium treated chickens.

The best feed conversion ratio was recorded, as expected, by the negative control group. However, the groups treated with H3 formula also had good feed conversion, similar to those of the uninfected chickens. The positive control group had the lowest use of feed.

**Fig. 2:**
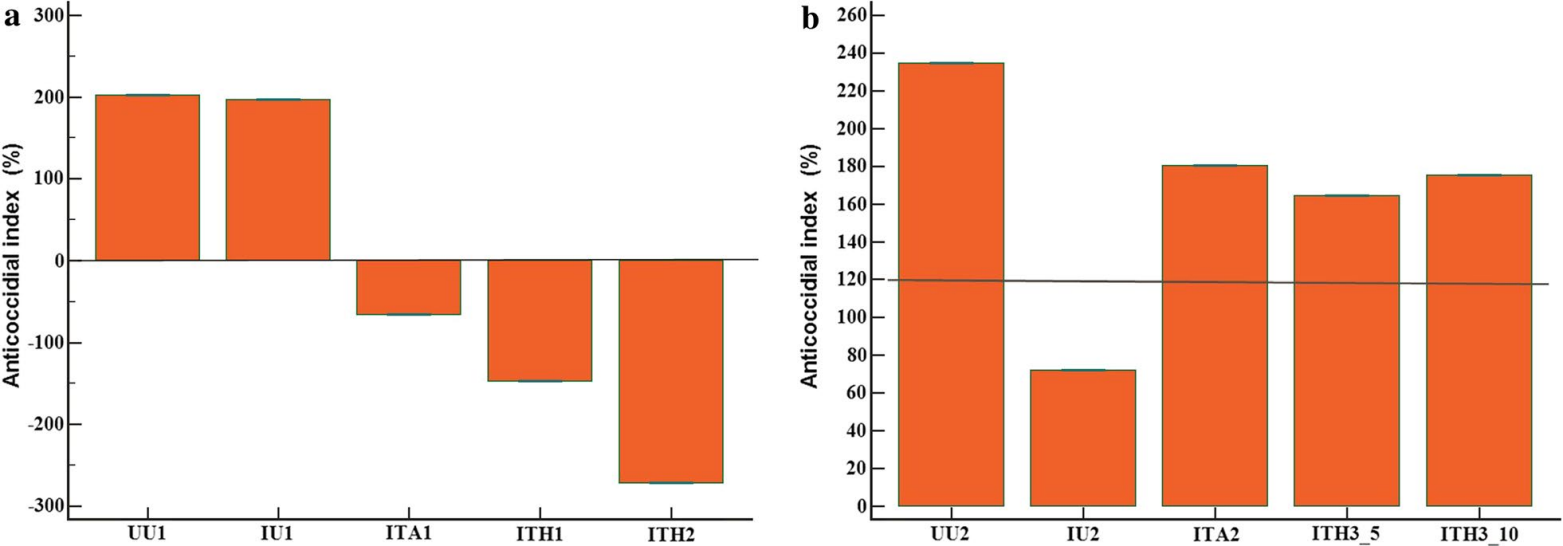
Anticoccidial index in experimental groups of chickens infected with *Eimeria* spp. (*E. acervulina*, *E. maxima* and *E. tenella*) and treated with the herbal product in different compositions compared with control (negative, positive and amprolium) groups. **a** First battery experiment when chickens were infected with 5 × 10^3^ sporulated oocysts of *Eimeria* spp. and treated with H1 and H2 formulas, 10 ml/l water. **b** Second battery experiment when chickens were infected with 5 × 10^4^ sporulated oocysts of *Eimeria* spp. and treated with H3 formula, 5 (ITH3-5) and 10 ml/l water (ITH310). The line at 120 represents the cut-off value for anticoccidian activity and values under the line means a lack of anticoccidian activity

The H3 formula was effective for control of experimental coccidiosis in chickens according to the anticoccidial index and it was not dose dependent (Fig. 2).

### Liquid chromatography tandem mass spectrometry

A total ion chromatogram and an extracted ion chromatogram of the H3 formula are presented in Additional file 1: Figure S1 and Additional file 2: Figure S2, respectively. The presence of seven flavonoids and polyphenols was tested (Table 2). The concentration of polyphenols was the highest, the sum of chlorogenic acid and caffeic acid being 914.9 µg/ml.

**Table 2:** Concentration of the tested compounds in the H3 formula

Compound	Concentration (µg/ml)	Amount (µg) in 1 l of water (H3a)	Amount (µg) in 1 l of water (H3b)
Apigenin	1.04	10.4	5.2
Kaempferol	0.23	2.3	1.1
Quercetin	0.29	2.9	1.4
Luteolin	4.93	49.3	24.6
Quercitrin	0.62	6.2	3.1
Chlorogenic acid	75.87	758.7	379.3
Caffeic acid	15.62	156.2	78.1

## Discussion

Herbal remedies have been used since ancient times in medicine and have recently gained increasing popularity, especially because of the declining effectiveness of synthetic compounds and concerns of the general population about drug side effects and interactions [18]. In chicken coccidiosis, herbal extracts have been intensively studied in the recent years in the search for new alternatives to the traditional anticoccidial drugs [27]. The extensive use of anticoccidials in the poultry industry may lead to the occurrence of drug residues in meat and eggs [32]. As such, consumer interest in organic foods has been rapidly increasing in recent years. The organic requirements restrict the use of chemicals, so natural plant products may represent an effective solution for pathogen control in the organic poultry system [28].

In this frame, the present study aimed to assess the anticoccidial effect of a natural plant product in three different compositions. H1 formula, which contained garlic and wild thyme extract, totally reduced the lesions produced by *E. acervulina*. H2 formula, which was comprised of oregano, summer savory and greater celandine, reduced the lesions caused by *E. tenella.* However, the chickens medicated with these formulas presented higher OPG output compared with the positive control group. This aspect can be explained by the over-multiplication of the uninhibited *Eimeria* species. According to Dar et al. [33], garlic administration increases the values of serum albumin, globulin and total proteins due to its anti-inflammatory and immunomodulatory action that repair the organ lesions induced by *Eimeria*, an aspect observed in the present study for the duodenum. Arczewska-Włosek and Świątkiewicz [26] observed an increased weight gain and better feed conversion in the chickens highly infected with *Eimeria* spp. whose diet was supplemented with garlic extract at a level of 750 mg/kg feed. Similar to our results, the OPG was higher than in the case of the infected unmedicated group. The authors stated that the high production performances, despite of the greater number of oocysts recorded, could be the effect of reduced damage to intestinal cells or promotion of enterocyte renewal, which can provide the substrate for coccidia multiplication. However, in our study the first two formulas of the commercial herbal formula did not improve the chickens’ production performance. Numerous studies have demonstrated that *Origanum vulgare* as aqueous extract at a concentration of 2 g/kg feed [34] or essential oil in concentrations of 600 and 1200 mg/kg feed [35] and also *Satureja hortensis* powder at 5 g/kg feed [36], or 1% powder in feed [37] stimulates the food intake and growth of chickens. However, as in the present study, Bozkurt et al. [38] showed that the administration of an essential oil blend that contained carvacrol, 1,8-cineole, camphor, and thymol derived from oregano, laurel leaf and lavender oil did not significantly improve the broiler growth performance. The authors concluded that the magnitude of improvement in weight gain also depends on other factors like gut microflora, mucus production or host immune response, which consume part of the nutrients used for growth. In the present study, because the herbs did not present anticoccidial activity on all *Eimeria* species used, the coccidian infection most probably invalidated the growth promoting effects of the plants. Kim et al. [24] found that garlic metabolites enhance chickens’ production performances and reduce the oocyst output in chickens challenged with *E. acervulina*, due to a direct cytotoxic effect on the coccidian sporozoites. This probably also occurred in our study based on the absence of *E. acervulina* lesions in the chickens medicated with the first formula. There are also studies that demonstrate the anticoccidial effect of *Origanum vulgare* essential oil on *E. tenella* at a level of 300 mg/kg feed [12]. In the present study the formula which contained oregano (H2) also had a good effect on reducing the lesions produced by *E. tenella*. A herbal extract that contained *Allium sativum*, *Salvia officinalis*, *Echinacea purpurea*, *Thymus vulgaris* and *Origanum vulgare* improved the performance parameters of broilers and reduced the oocyst output [39]. It seems that in a mixed coccidian infection, the combination of different herbs may represent the solution in controlling the disease.

As shown in the present study, the multi-herb product H3 highly reduced the coccidian multiplication rate and reduced the severity of intestinal lesions. The product had a lower anticoccidial effect on *E. acervulina*, but a good effect on *E. tenella* (markedly reduced OPG output and medium reduced lesion score). The synergistic effects of the combined herbs enhanced the anticoccidial activity of broiler chickens medicated with H3. Some of the herbs from the mixed extract already have proven anticoccidial activity such as *Allium sativum* powder, supplemented in broiler feed at 0.1% [13] and *Thymus serpyllum* in ducks at a concentration of 2,500 mg/kg [40]. These two herbs were included in the 1st tested herbal formula. Other herbs used in H3 composition like *Urtica dioica*, *Rosmarinus officinalis*, *Tanacetum vulgare*, *Coriandrum sativum* or *Glycyrrhiza glabra* are known to have immunomodulatory effects [33, 41–44]. Moreover, many of these plants contain flavonoids, tannins or saponins that act as antioxidants which reduce the oxidative stress caused by reactive oxygen species encountered also in coccidiosis [27]. The antioxidant capacity of a herbal product is directly linked with its anticoccidial effect [45]. As shown in LC-MS/MS, the H3 formula is a rich source of polyphenols. The chlorogenic acid was in high concentration, followed by the caffeic acid and the luteolin. Chlorogenic acid was found to have antibacterial and antibiofilm properties against nosocomial pathogen strains [46]. Furthermore, chlorogenic acid and caffeic acid are powerful antioxidants [47], which neutralize the reactive oxygen species that are produced during *Eimeria* infection, as stated above. The antioxidants can alleviate the damage to the intestinal tissue during parasite invasion by reducing the cytotoxic effects caused by the reactive oxygen species [48], and thus can explain the lower lesion score observed in the chickens treated with H3 formula.

## Conclusions

H3 formula was effective in controlling experimental coccidiosis in chickens and can be used successfully as a natural anticoccidial. Field trials are, however, recommended in order to validate the data obtained in experimental studies.

## Additional files

**Additional file 1: Figure S1.** Total ion chromatogram of the H3 formula.

**Additional file 2: Figure S2.** Extracted ion chromatogram of the H3 formula.

## Data Availability

The datasets used and/or analysed during the present study are available from the corresponding author upon reasonable request.

## Abbreviations

H: herbal formula
H1: first herbal formula
H2: second herbal formula
H3: third herbal formula
BE: battery experiment
BE1: first battery experiment
BE2: second battery experiment
UU1: uninfected untreated control in BE1
UU2: uninfected untreated control in BE2
IU1: infected untreated control in BE1
IU2: infected untreated control in BE2
ITA1: infected treated with amprolium in BE1
ITA2: infected treated with amprolium in BE2
ITH1: infected treated with H1 10 ml/l water in BE1
ITH2: infected treated with H2 10 ml/l water in BE1
ITH3-5: infected treated with H3 5 ml/l water in BE2
ITH3-10: infected treated with H3 10 ml/l water in BE2
PCR: polymerase chain reaction
LC-MS/MS: liquid chromatography-tandem mass spectrometry
SRM: multiple reactions monitoring mode
OPG: oocysts shed per gram of feces
LS: lesion score
MR: mortality rate
WG: weight gain
FCR: feed conversion ratio
ACI: anticoccidial index
p.i.: post-infection
%S: percentage survival
%RGW: percentage of relative weight gain
LI: the lesion index
OI: oocyst index
