# Supplementary figures and images for: Efficacy of a commercial herbal formula in chicken experimental coccidiosis
Source: Parasit Vectors. 2019 Jul 12;12:343. doi: 10.1186/s13071-019-3595-4 (PMC6624883; doi:10.1186/s13071-019-3595-4)

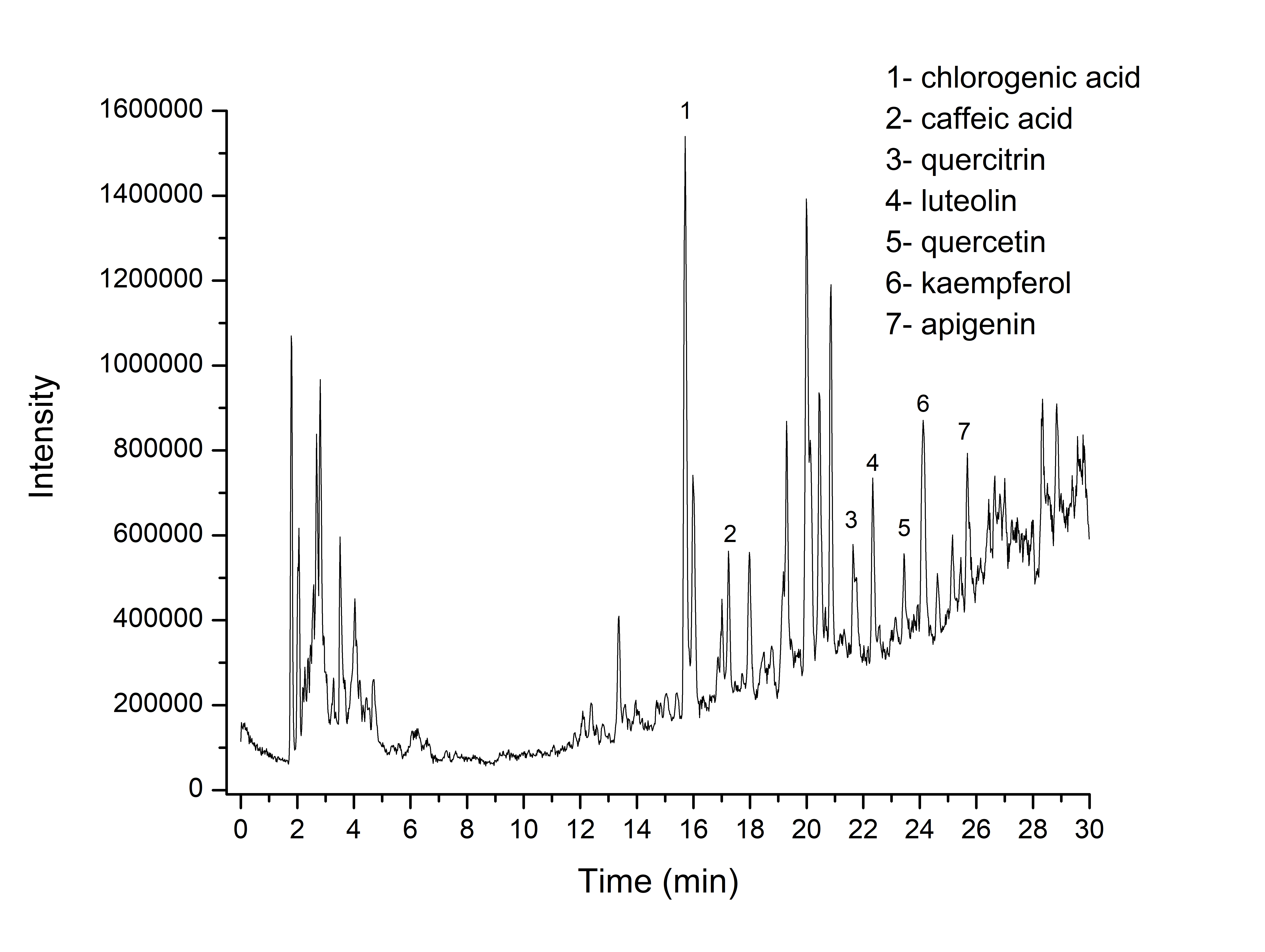

Supplement: Supplementary file 1 — Additional file 1: Figure S1. Total ion chromatogram of the H3 formula. [file 13071_2019_3595_MOESM1_ESM.jpg]

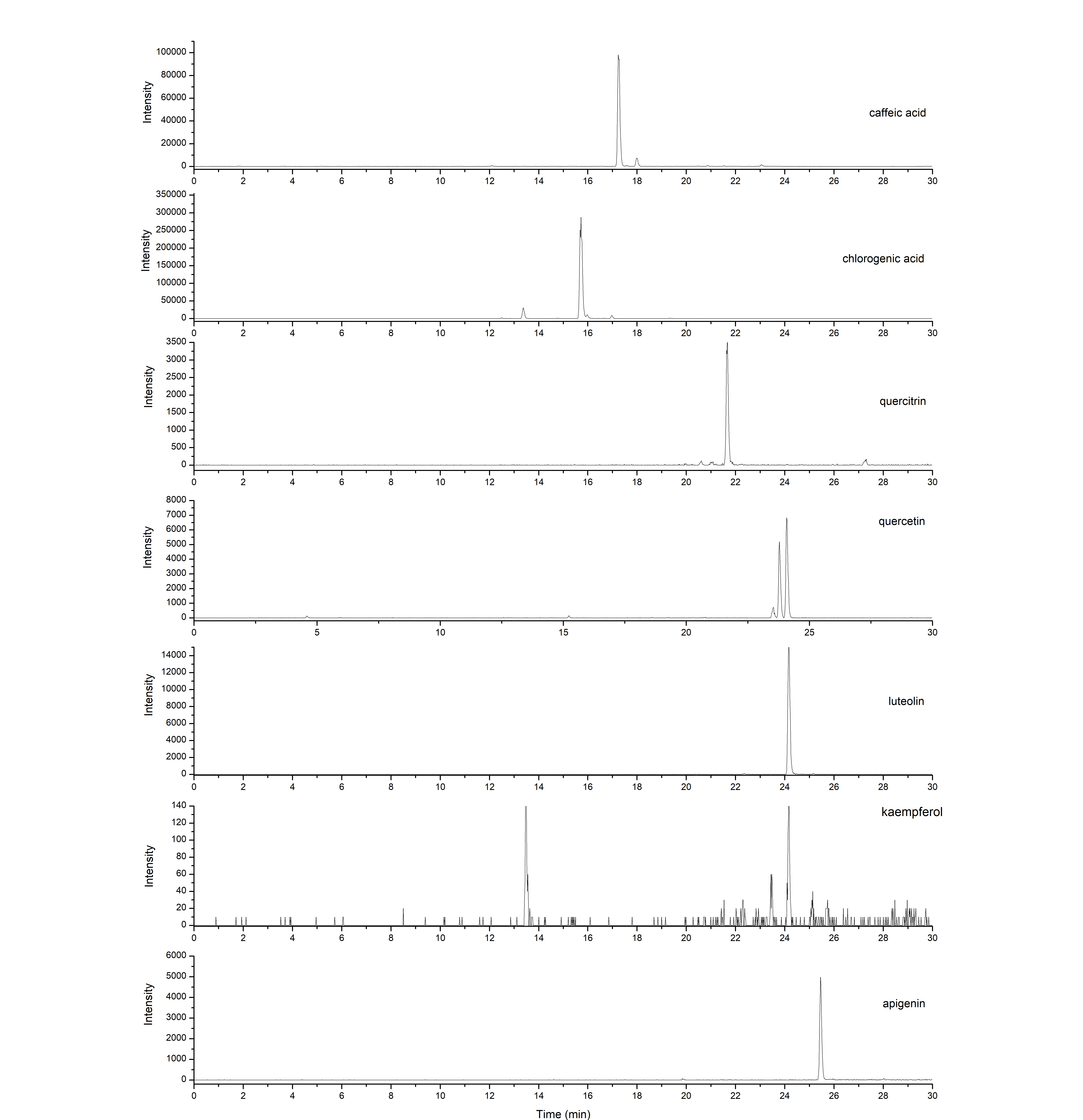

Supplement: Supplementary file 2 — Additional file 2: Figure S2. Extracted ion chromatogram of the H3 formula. [file 13071_2019_3595_MOESM2_ESM.jpg]
